# Double-Stranded RNA-Degrading Enzymes Reduce the Efficiency of RNA Interference in *Plutella xylostella*

**DOI:** 10.3390/insects12080712

**Published:** 2021-08-09

**Authors:** Jin-Zhi Chen, Ying-Xia Jiang, Miao-Wen Li, Jian-Wen Li, Ben-Hu Zha, Guang Yang

**Affiliations:** 1 State Key Laboratory of Ecological Pest Control for Fujian and Taiwan Crops, Institute of Applied Ecology, Fujian Agriculture and Forestry University, Fuzhou 350002, China; chjinzhi@yeah.net (J.-Z.C.); jyx18270821925@163.com (Y.-X.J.); miaowenLee@163.com (M.-W.L.); li1928999798@163.com (J.-W.L.); benhu_zha@163.com (B.-H.Z.); 2 Joint International Research Laboratory of Ecological Pest Control, Ministry of Education, Fuzhou 350002, China; 3 Key Laboratory of Integrated Pest Management for Fujian-Taiwan Crops, Ministry of Agriculture and Rural Affairs, Fuzhou 350002, China; 4 Key Laboratory of Green Control of Insect Pests (Fujian Agriculture and Forestry University), Fujian Province University, Fuzhou 350002, China; 5 Ministerial and Provincial Joint Innovation Centre for Safety Production of Cross-Strait Crops, Fujian Agriculture and Forestry University, Fuzhou 350002, China; 6 College of Plant Protection, Fujian Agriculture and Forestry University, 15 Shangxiadian Road, Cangshan, Fuzhou 350002, China

**Keywords:** dsRNase, RNA interference, diamondback moth, RNA degradation

## Abstract

**Simple Summary:**

The efficiency of Lepidoptera RNA interference (RNAi) is highly varied among different species, different periods, and different genes. The stability of dsRNA is one of the important factors. DsRNA-degrading enzymes (dsRNases) are the key factors affecting the stability of dsRNA in insects. The efficiency of RNAi in diamondback moths was low and unstable. Furthermore, in vitro experiments, we found that dsRNA was completely degraded when incubated with the hemolymph or gut fluid of diamondback moths. Therefore, we hypothesized that the efficiency of RNAi in diamondback moths was decreased predominantly due to degradation of dsRNA by dsRNase. In this study, we identified four dsRNases in diamondback moths: *PxdsRNase1* was mainly expressed in the hemolymph; and *PxdsRNase2* and *PxdsRNase3* were mainly expressed in the intestinal tract. PxdsRNase1, PxdsRNase2, and PxdsRNase3 were verified to be involved in the RNAi process in diamondback moths. In vitro, the recombinant protein of PxdsRNase1 degraded dsRNA completely and PxdsRNase3 cleaved dsRNA without complete degradation. Overall, our findings provided a fundamental basis for understanding the mechanism of dsRNase involvement in the RNAi process and using RNAi to control diamondback moths in the future.

**Abstract:**

DsRNA-degrading enzymes (dsRNases) have been recognized as important factors in reducing RNA interference (RNAi) efficiency in different insect species. However, dsRNases in *Plutella xylostella* are still unknown. We identified the full-length cDNAs of *PxdsRNase1*, *PxdsRNase2*, *PxdsRNase3*, and *PxdsRNase4*. Gene expression profile showed that *PxdsRNase1* was mainly expressed in the hemolymph; and that *PxdsRNase2* and *PxdsRNase3* were mainly expressed in the intestinal tract. The expression of *PxCht* (*Chitinase* of *P. xylostella*) in *P. xylostella* larvae injected with the mixture of dsPxCht (dsRNA of *PxCht*) and dsPxdsRNase1 (dsRNA of *PxdsRNase1*), dsPxdsRNase2 (dsRNA of *PxdsRNase2*), or dsPxdsRNase3 (dsRNA of *PxdsRNase3*) was significantly higher than that in the larvae injected with the mixture of dsGFP (dsRNA of green fluorescent protein gene, *GFP*) and dsPxCht; the transcription level of *PxCht* in the larvae feeding on the mixture of dsPxCht and dsPxdsRNase1, dsPxdsRNase2, or dsPxdsRNase3 was significantly higher than that in the larvae feeding on the mixture of dsPxCht and dsGFP. The recombinant protein of PxdsRNase1 degraded dsRNA rapidly, PxdsRNase3 cleaved dsRNA without complete degradation, and PxdsRNase2 could not degrade dsRNA in vitro. These results suggested that *PxdsRNases1*, *PxdsRNases2*, and *PxdsRNases3* were involved in the dsRNA degradation to reduce RNAi efficiency with different mechanisms.

## 1. Introduction

The efficiency of Lepidoptera RNAi is highly varied among different species, different periods, and different genes [1]. The dsRNA degradation [2,3,4] or the inability of dsRNA to enter the cytoplasm [3,5,6] are the main factors affecting RNAi efficiency. Studies have also shown that dsRNA needs to last long enough in the midgut or hemolymph to be absorbed into cells to produce an effective RNAi response [7]. DsRNases are the key factors affecting the stability of dsRNA in insects. The activity of dsRNase has been observed in several insects. In *Bombyx mori*, dsRNase is expressed in the digestive juice and midgut, then secreted into the intestinal cavity for nucleic acid digestion [8,9]. Subsequently, dsRNases are found in more and more insects, such as *Lygus lineolaris* [10], *Manduca sexta* [11], *Acyrthosiphon pisum* [12], *Schistocerca gregaria* [13], and *Spodoptera frugiperda* [14].

In the hemolymph of *M. sexta*, dsRNA is degraded within 1 h, which proves the presence of dsRNase [11]. The RNAi efficiency is increased after the expression of dsRNase in *Locust migratoria* and *S. gregaria* is suppressed [15], verifying that the RNAi efficiency could be affected by dsRNase in insects. Furthermore, in *Anthonomus grandis*, the RNAi sensitivity is increased after dsRNA of nuclease is ingested to suppress the nuclease activity [16]. Interestingly, multiple dsRNases working together could enhance their effect on RNAi efficiency in *Spodoptera litura* [17,18]. The RNAi efficiency is increased after knockdown of *RNAi efficiency-related nucleases* (*REases*) in *Ostrinia furnacalis*, and suppressed after up-regulation of *REase* in *Drosophila melanogaster* [19].

With in vitro experiments, we found that dsRNA was completely degraded when incubated with the hemolymph or gut fluid of *P. xylostella*. Therefore, we hypothesized that the efficiency of RNAi in *P. xylostella* was decreased predominantly due to degradation of dsRNA by dsRNase. To verify this hypothesis, we performed a genome-wide search to identify genes encoding dsRNases in the *P. xylostella* and conducted function analysis of these dsRNases to understand the role of dsRNases in the RNAi process in *P. xylostella*.

## 2. Materials and Methods

### 2.1. Insect Rearing

The *P. xylostella* Fuzhou-sensitive strain (FZss) used in the experiment was maintained at the Institute of Applied Ecology, Fujian Agriculture and Forestry University, Fuzhou, China. The colony was maintained on radish (*Raphanus sativus*) seedlings without exposure to any chemical insecticide at 25 °C with the photoperiod of 16L: 8D and 60–70% relative humidity (RH) in the growth chamber.

The *P. xylostella* sensitive strain (SLss) was reared by the artificial diet [20]. Pupae were collected in a paper cup with a cotton wool containing 10% honey as extra nutrition for adults, and eggs were collected by a parafilm card stained with vegetable powder hung in the cup. The insect was reared at 26 ± 1 °C, 60–80% RH, and the photoperiod of 16L: 8D.

### 2.2. Isolation and Sequencing of PxdsRNase cDNAs

The *BmdsRNase* (dsRNase of *B. mori*, GenBank ID: NP_001091744.1) was used as a query in tBLASTn to search the genomic database of *P. xylostella* (http://59.79.254.1/DBM/blast.php (accessed on 24 October 2017)) for obtaining the cDNA sequence of *PxdsRNase*. Total RNA was extracted from the 4th-instar larvae using the RNA extraction kit (Promega, Madison, WI, USA), according to the manufacturer’s instructions. First-strand complementary DNA (cDNA) synthesis was performed from 1 μg of total RNA using the reverse transcription kit (Promega, Madison, WI, USA). PCR was performed with the cDNA template and gene-specific primers (Table 1) to amplify the full-length cDNA sequence of *PxdsRNase* in the following conditions: 95 °C for 5 min; 35 cycles of 95 °C for 30 s, 55 °C for 15 s, and 72 °C for 2 min; and 72 °C for 10 min. The PCR product was purified using the E.Z.N.A ^TM^ Gel Extraction kit (Omega, Doraville, GA, USA), inserted into the pESI-Blunt Zero plasmid (Yeasen, Shanghai, China), and then sequenced in two directions by Shangya Biological Company (Fuzhou, China).

### 2.3. Amino Acid Sequence Analysis of PxdsRNases

The cDNA sequences of *PxdsRNases* were translated into the amino acid sequence by the online tools available from the ExPASy website (https://web.expasy.org/translate/ (accessed on 16 November 2017)). Domain architecture and signal peptides were predicted by the SMART domain analysis (http://smart.embl-heidelberg.de/ (accessed on 16 November 2017)) and the SignalP 4.1 Server (http://www.cbs.dtu.dk/services/Sig-nalP/ (accessed on 16 November 2017)), respectively. Web BLAST tools (https://blast.ncbi.nlm.nih.gov/ (accessed on 16 November 2017)) were used to analyze the conserved regions, including the endonuclease NS domain, active site, Mg^2+^ binding site, and substrate binding site.

### 2.4. Determination of Gene Expression of PxdsRNase Genes in Different Tissues and Developmental Stages

For tissue-specific expression of *PxdsRNase* genes, the total RNA was extracted from the integument, fat body, gut, malpighian tubule, hemolymph, silk and head of 20 4th-instar larvae. For determining the gene expression in different developmental stages, the total RNA was extracted from eggs, 1st, 2nd, 3rd, 4th larvae, pupae and adults. The first-strand cDNA was synthesized from 1 μg of total RNA using the reverse transcription kit (Promega, Madison, WI, USA). *PxdsRNase*-, *PxCht*-, *EF1*-specific primers used for the quantitative PCR (qPCR) are shown in Table 1. Each 20 μL of qPCR mixture consisted of 10 μL SYBR ^TM^ Green Real-time PCR Master Mix (Progema, Madison, WI, USA), 2 μL of 10-fold diluted template cDNA, 0.4 μL of 10 μM of each primer, 0.15 μL of ROX and 7.05 μL of deionized water. The PCR reaction conditions were 95 °C for 10 min, 40 cycles of 95 °C for 15 s, and 60 °C for 30 s. A melt curve was developed to confirm the amplification specificity for each qPCR. Three technical replicates of each qPCR and three biological replicates of each treatment were conducted. Gene expression level was analyzed using the 2^−△CT^ method, and statistical analyses were performed using one-way ANOVA (analysis of variance) followed by the Turkey test (*p* < 0.05, SPSS software, SPSS Inc. Chicago, IL, USA).

### 2.5. In Vitro Incubation of dsRNA with Hemolymph/Gut Fluid

Hemolymph was collected with a capillary glass tube from the amputated legs of 30 4th-instar larvae, diluted with 50 μL of PBS, and then centrifuged at 16,000× *g* for 10 min to remove hemocytes. The supernatant was collected and stored at −20 °C. Meanwhile, guts from 30 larvae of *P. xylostella* were dissected and collected in cold 1.5 mL tubes with 50 μL of 1× PBS buffer, and centrifuged at 16,000× *g* for 10 min. The supernatant was collected and stored at −20 °C.

The total protein concentration of the hemolymph or gut fluid was examined using the BCA Protein Quantification kit (Yesea, Shanghai, China), according to the manufacturer’s instruction. Measurements were performed in the Synergy Mx microplate reader (BioTek, Doraville, GA, USA).

For an in vitro incubation assay, 1 μL of dsPxCht solution (containing 120 ng of dsRNA, 564 bp) was mixed with 10 μg of total protein of hemolymph or gut fluid in 1.5 mL microcentrifuge tube and incubated at 28 °C for 0 min, 5 min, 30 min, 60 min, 360 min, and 600 min, separately. Next, 120 ng of dsCht was mixed with 1 μg, 5 μg, 10 μg, 20 μg, and 30 μg of total protein of hemolymph or gut fluid in 1.5-mL microcentrifuge tube and incubated at 28 °C for 5 min. After incubation, these samples were mixed with 1 μL of loading buffer and checked in 1% agarose gel. The gel was visualized using a UV transilluminator (Vilber, Pairs, France) to analyze the integrity of dsRNA.

### 2.6. RNAi Response after dsRNA Injection or Oral Delivery

DsRNAs were prepared in vitro by the T7 RiboMAX Express RNAi System (Promega, USA). Primers (Table 1) for dsRNA synthesis of *PxCht* (564 bp), *GFP* (417 bp) and *PxdsRNases* (407 bp of *PxdsRNases1*, 476 bp of *PxdsRNases2*, 458 bp of *PxdsRNases3*, and 412 bp of *PxdsRNases4*) were designed using the NCBI web service (https://www.ncbi.nlm.nih.gov/tools/primer-blast/ (accessed on 8 May 2018)). The templates containing the T7 promoter sequence at both ends were synthesized through PCR by 2× Taq Master Mix (Vazyme, Nanjing, China). The PCR was performed at the condition: 94 °C for 5 min; 35 cycles of 95 °C for 30 s, 55 °C for 30 s, and 72 °C for 40 s; and 72 °C for 10 min. The PCR products were purified using the E.Z.N.A ^TM^ Gel Extraction Kit (Omega, Doraville, GA, USA). About 2 μg of each purified PCR product was used to synthesize dsRNA, dsRNA was dissolved with 20 μL of nuclease-free water, and the final concentration of dsRNA was adjusted to 4.0 μg/μL.

To compare the RNAi responses induced by dsRNA between injection and oral delivery, *PxCht* involved in the molting process was selected as the RNAi target gene. DsGFP, dsPxdsRNase, the mixture of dsGFP and dsPxCht, and the mixture of dsPxCht and dsPxdsRNase were separately injected into the internode of abdomen of one 4th-instar larva of FZss at the amount of 600 ng by a microsyringe and separately fed to the 100 larvae at the amount of 3 μg. All the treated larvae were reared in the same condition, as previously described. The total RNA of 5 larvae were extracted for each treatment, and RT-qPCR was performed with the primers listed in Table 1 to determine the expression of *PxCht* and *PxdsRNase* using *EF1* as the reference gene. Three replications were performed for each treatment. The Turkey test after one-way ANOVA was used for determining differences in RNAi efficiency among different treatments.

### 2.7. Heterologous Expression of PxdsRNases

The codon-optimized cDNA sequences of *PxdsRNase1*, *PxdsRNase2*, and *PxdsRNase3* without the signal peptide and with 6× His tags attached at its 3′ end, were individually inserted at *Ndel*I/*Hind*III in PET-30a(+) vector by the Genscript Biotech Corporation (Nanjing, China). The constructed plasmids were individually transformed into *E. coli* DH5α competent cells, and the subsequent cells were cultured at 37 °C for 14 h. Positive clones were picked and verified by PCR and sequencing.

The recombinant plasmid extracted from the transformed *E. coli* DH5α was transformed into *E. coli* BL21 (DE3). Then, the dsRNases were expressed by the method of He et al. [21], and extracted by the ultrasonically crushing method [22]. The extracted proteins were identified by SDS-PAGE and Western blot.

### 2.8. Determination of PxdsRNase Activity

The protein concentration was determined as the method described in 2.5. To test the dsRNA-degrading activity of PxdsRNase, 1 μg dsRNA dissolved in 5 μL of nuclease-free water was added to 20 μL of recombinant enzyme solution of PxdsRNase1 (1 μg, 2 μg, 3 μg, 4 μg, and 5 μg), PxdsRNase2 (5 μg, 10 μg, 15 μg, 20 μg, and 30 μg), or PxdsRNase3 (1 μg, 2 μg, 3 μg, 4 μg, and 5 μg), and then incubated at 28 °C for 15 min. After incubation, the samples were examined as the method described in Section 2.5.

## 3. Results

### 3.1. Identification of DsRNases in P. xylostella

**Figure 1:**
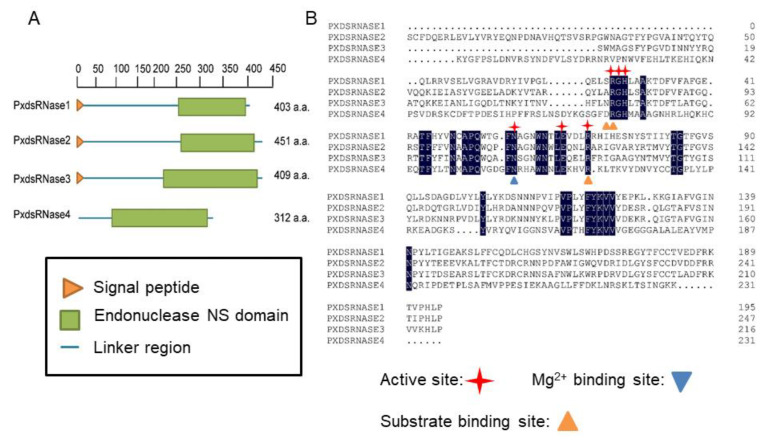
Analysis of deduced amino acid sequences of *PxdsRNase* genes. (**A**) Schematic diagram of deduced amino acid domains of PxdsRNase1, PxdsRNase2, PxdsRNase3 and PxdsRNase4. An orange triangle stands for the location of signal peptide, green boxes endonuclease NS domains, and blue lines linker regions. (**B**) Multiple sequence alignments of endonuclease NS domain of deduced dsRNase amino acid sequences in *P. xylostella*.

Four cDNA sequences putatively encoding *PxdsRNase1* (GenBank: MZ517187), *PxdsRNase2* (GenBank: MZ517188), *PxdsRNase3* (GenBank: MZ517189), and *PxdsRNase4* (GenBank: MZ517190) were identified from *P. xylostella* genome. The cDNA sequences were cloned by RT-PCR and sequenced. The ORFs of these four genes were of 1212 bp, 1359 bp, 1350 bp and 939 bp, encoding 403, 451, 449 and 312 amino acids, respectively. The domain analyses showed that all enzymes contained an endonuclease NS domain and a signal peptide, except PxdsRNase4 which did not have a signal peptide (Figure 1A). Alignment of the endonuclease domains of PxdsRNases indicated that their amino acid sequences were of high identity, with six active sites, three substrate binding sites, and an Mg^2+^ binding site (Figure 1B).

### 3.2. Stage-Specific and Tissue-Specific Expression of PxdsRNase Genes

**Figure 2:**
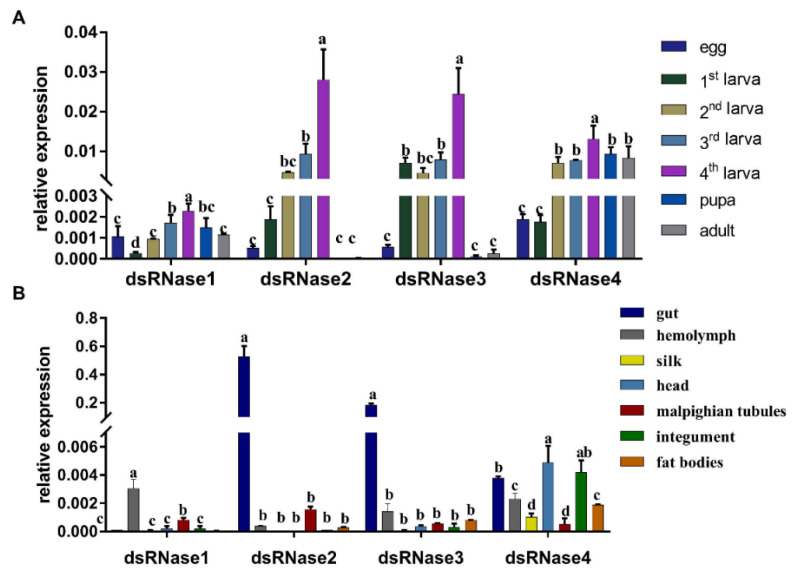
Relative expression levels of *PxdsRNase* genes. (**A**) Relative expression levels of *PxdsRNase* genes in different stages; (**B**) Relative expression levels of *PxdsRNase* genes in different tissues of the fourth instar larvae. *EF1* was used as the reference gene. One-way ANOVA was conducted to calculate the statistical significance, followed by the Turkey test. Different letters on the bars represent significant differences (*p* < 0.05) among different samples.

In general, *PxdsRNase* genes had the highest expression levels of mRNA in the fourth instar larvae; *PxdsRNase1* had a lower expression level compared with other three *PxdsRNase* genes in *P. xylostella* (Figure 2A). In different stages, *PxdsRNase2* and *PxdsRNase3* exhibited high expression in the second–fourth instar larvae; and *PxdsRNase4* had high expression from the second instar larva to adult (Figure 2A). In different tissues, *PxdsRNase1* had the highest expression level in the hemolymph; *PxdsRNase2* and *PxdsRNase3* were almost exclusively expressed in the gut with a higher level of *PxdsRNase2* than *PxdsRNase3*; and *PxdsRNase4* had high expression level in the head, integument, and gut (Figure 2B).

### 3.3. DsRNA Degradation by the Proteins Extracted from the Gut and Hemolymph of Larvae

**Figure 3:**
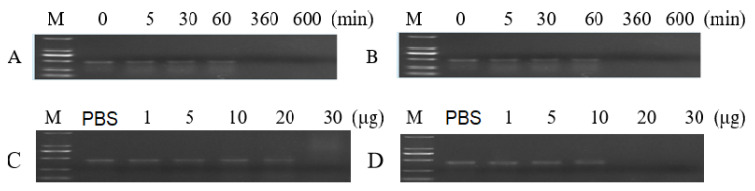
Degradation of dsRNA by the total proteins extracted from the gut or hemolymph at 28 °C. (**A**) Incubation of 120 ng dsRNA with 10 μg of total gut proteins for different times. (**B**) Incubation of 120 ng dsRNA with 10 μg of total hemolymph proteins for different times. (**C**) Incubation of 120 ng dsRNA with 1–30 μg of total gut proteins for 5 min. (**D**) Incubation of 120 ng dsRNA with 1–30 μg of total hemolymph proteins for 5 min.

DsRNA of 120 ng was totally degraded by 10 μg of total proteins extracted from the gut or hemolymph in 6 h at 28 °C (Figure 3A,B), indicating that the enzymes in the gut or hemolymph could degrade the dsRNA. The total proteins of 30 μg extracted from the gut and 20 μg of total proteins extracted from the hemolymph degraded 120 ng of dsRNA in 15 min at 28 °C, individually, which indicated that the hemolymph proteins might have a higher ability to degrade dsRNA than the gut proteins (Figure 3C,D).

### 3.4. Effects of PxdsRNase Suppression on RNAi Efficiency

**Figure 4:**
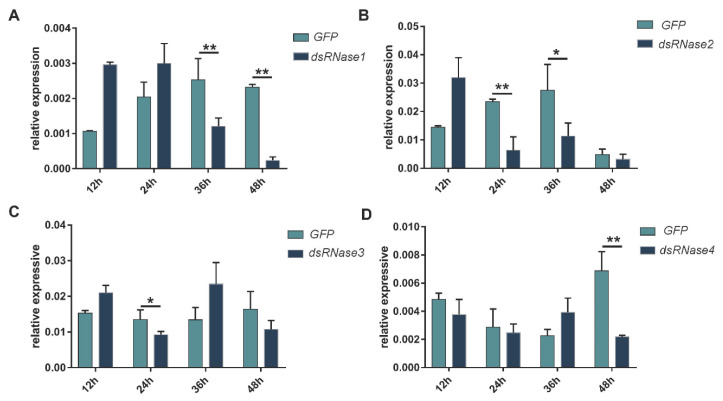
Effects of dsRNA injection on the expression levels of *PxdsRNases*. The fourth instar larvae were injected with 600 ng of different dsPxdsRNases or dsGFP, and the transcription levels of *PxdsRNase* genes were detected by RT-qPCR at different times. *EF1* was used as the reference gene for normalization. Statistical analyses were performed using one-way ANOVA followed by the Turkey test. (**A**) *PxdsRNase1*; (**B**) *PxdsRNase2*; (**C**) *PxdsRNase3*; (**D**) *PxdsRNase4*. (*, *p* < 0.05; **, *p* < 0.01).

**Figure 5:**
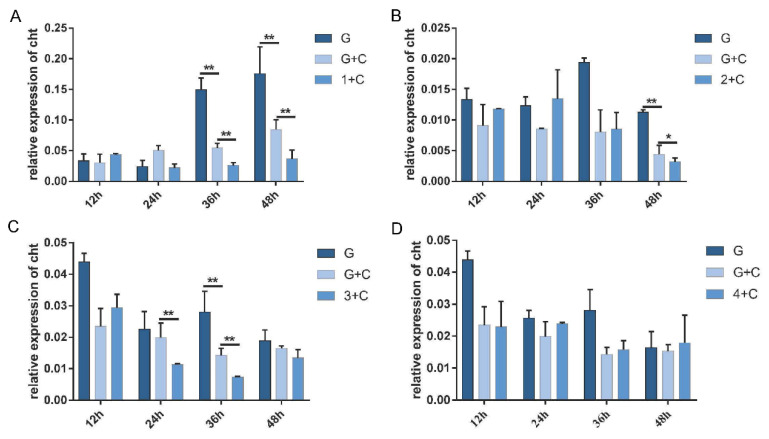
RNAi efficiency of *PxCht* after RNAi of *PxdsRNase* by dsRNA injection. RNAi efficiency of *PxCht* in the f instar larvae after injection with 1200 ng of dsGFP, a mixture of ds PxCht and dsGFP, or a mixture of dsPxdsRNase and dsPxcht, was evaluated by RT-qPCR, and *EF1* was used as the reference gene for normalization. Statistical analyses were performed using one-way ANOVA followed by the Turkey test. G, dsGFP; C, dsPxCht; 1, dsPxdsRNase1; 2, dsPxdsRNase2; 3, dsPxdsRNase3; and 4, dsPxdsRNase4. (**A**) *PxdsRNase1*; (**B**) *PxdsRNase2*; (**C**) *PxdsRNase3*; (**D**) *PxdsRNase4*. (*, *p* < 0.05; **, *p* < 0.01).

In the injection experiment, the expression levels of *PxdsRNase1* were significantly suppressed by dsRNA at 36 h and 48 h, *PxdsRNase2* at 24 h and 36 h, *PxdsRNase3* at 24 h, and *PxdsRNase4* at 48 h (Figure 4). Co-injection of dsPxdsRNase1/dsPxdsRNase2/dsPxdsRNase3 + dsPxCht (1/2/3 + C) caused significant reduction in the transcript levels of *PxCht* compared with dsGFP +dsPxCht (G + C) (Figure 5A–C), but not with dsPxdsRNase4 + dsPxCht (4 + C) (Figure 5D). Therefore, RNAi efficiency in *P. xylostella* was improved after suppression of *PxdsRNas1*, *PxdsRNas2*, or *PxdsRNas3* by dsRNA injection.

**Figure 6:**
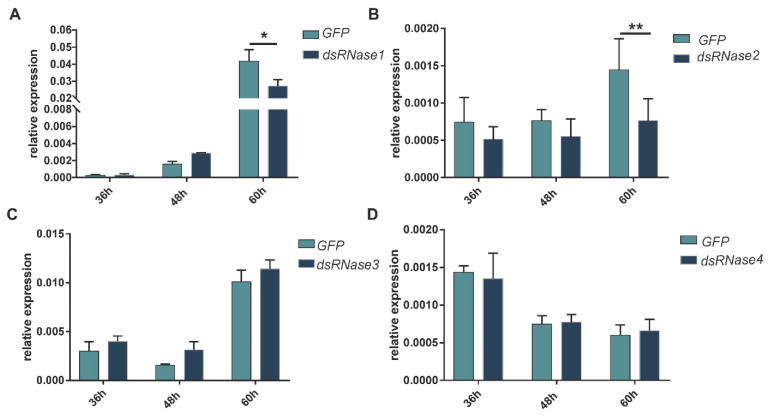
Effects of uptake dsRNA on relative expression levels of *PxdsRNase* genes in the larvae. Three μg of dsPxdsRNase (dsPxdsRNase1, dsPxdsRNase2, dsPxdsRNase3 or dsPxdsRNase4) or dsGFP were fed to the fourth instar larvae, and the transcription levels of *PxdsRNase* genes were measured by RT-qPCR. *EF1* was used as the reference gene for normalization. Statistical analyses were performed using one-way ANOVA followed by the Turkey test. (**A**) *PxdsRNase1*; (**B**) *PxdsRNase2*; (**C**) *PxdsRNase3*; (**D**) *PxdsRNase4*. (*, *p* < 0.05; **, *p* < 0.01).

**Figure 7:**
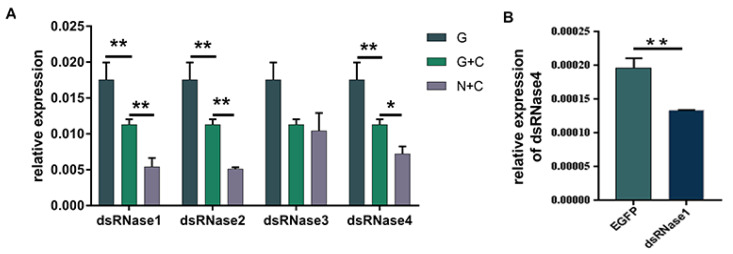
RNAi efficiency of *PxCht* after RNAi of *PxdsRNase* by oral dsRNA. (**A**) Expression level of *PxCht* in the fourth instar larvae 60 h after oral administration of 1200 ng of dsRNA of *GFP*, a mixture of dsGFP and dsPxCht (G + C) or a mixture of dsPxdsRNases and dsPxCht (N + C) was evaluated by RT-qPCR, and *EF1* was used as the reference gene for normalization. Statistical analyses were performed using one-way ANOVA followed by the Turkey test. G, dsGFP; C, dsPxCht; N, dsPxdsRNase1/ dsPxdsRNase2/ dsPxdsRNase3/ dsPxdsRNase4. (**B**) *PxdsRNase1* relative expression level in the fourth instar larvae 60 h after oral administration of dsPxdsRNase4. (*, *p* < 0.05; **, *p* < 0.01).

In the feeding experiment, the expression level of *PxdsRNas1* in the larvae showed a significant reduction at 60 h post-feeding on dsPxdsRNase1 (Figure 6A), as well as *PxdsRNase2* (Figure 6B). Interestingly, there was no suppression observed by dsPxdsRNase3 (Figure 6C) and dsPxdsRNase4 (Figure 6D). The expression level of *PxCht* in the larvae feeding on dsPxdsRNase1 + dsPxCht (1 + C) was significantly lower than that in the larvae feeding on dsGFP+ dsPxCht (G + C), and the same case for dsPxdsRNase2 + dsPxCht (2 + C) and dsPxdsRNase4 + dsPxCht (4 + C) (Figure 7A). The expression level of *PxCht* in the larvae feeding on dsPxdsRNase3 + dsPxCht (3 + C) was not changed compared with that in the larvae feeding on dsGFP+ dsPxCht (G + C) (Figure 7A). The expression of *PxdsRNase1* was observed to be significantly suppressed by dsPxdsRNase4 (Figure 7B). Therefore, oral administration of dsPxdsRNase4 might increase the RNAi efficiency by suppressing *PxdsRNase1*. The above results indicated that the RNAi efficiency in *P. xylostella* was enhanced after suppression of *PxdsRNase1* and *PxdsRNase2*.

### 3.5. Enzymatic Activities of PxdsRNases

**Figure 8:**
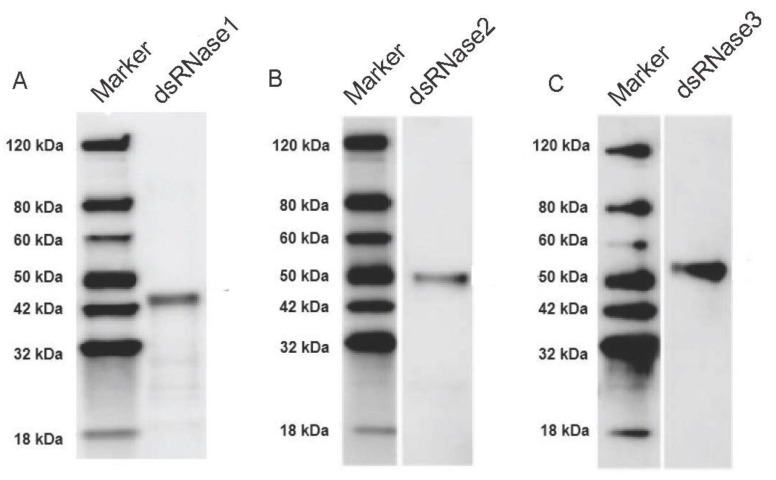
Western blot analysis of recombinant PxdsRNase proteins. Electrophoresis was performed in 12% SDS-PAGE gel. The separated protein was transferred to membrane (100 V for 100 min). After blocking with 5% *w*/*v* BSA (Solarbio, Beijing, China), each protein was incubated with His monoclonal antibody (GenScript, Nanjing, China), separately. The EstinTM L1 staining kit (GenScript, Nanjing, China) was used for signal generation. The arrows point to the target proteins. (**A**) PxdsRNase1; (**B**) PxdsRNase2; (**C**) PxdsRNase3.

**Figure 9:**
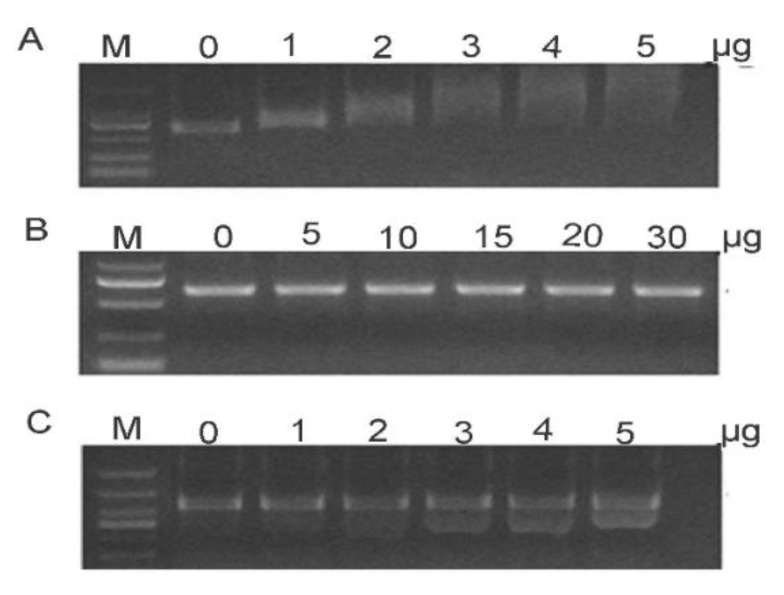
Degradation of dsRNA by recombinant PxdsRNases. M, marker. (**A**) PxdsRNase1; (**B**) PxdsRNase2; (**C**) PxdsRNase3.

The recombinant proteins, His-PxdsRNase1, His-PxdsRNase2, and His-PxdsRNase3, were successfully expressed in the prokaryotic expression system with the estimated molecular masses of 43 kDa, 50 kDa, and 51 kDa, respectively (Figure 8), which were consistent with the expected protein sizes determined by the sequences. PxdsRNase1 showed a high dsRNase activity to degrade dsPxCht (Figure 9A), PxdsRNase2 had no effect on dsRNA (Figure 9B), and PxdsRNase3 cleaved dsRNA without complete degradation (Figure 9C).

## 4. Discussion

DsRNases are widely distributed in different organisms. The number of dsRNases varies from one to five among insects, such as, four dsRNases in *L. migratoria* and *S. gregaria* [13,15], three dsRNases in *A. grandis* [16], five dsRNases in *S. litura* and *T. castaneum* [18,23]. In *P. xylostella*, 4 dsRNases, *PxdsRNase1*, *PxdsRNase2*, *PxdsRNase3* and *PxdsRNase4*, were identified. There is still no evidence to demonstrate that more dsRNases cause lower RNAi efficiency in one species. PxdsRNases shared similar conserved domains of endounuclease NS domain active site and substrate binding site with dsRNases in *B. mori*, *L. migratoria*, and *S. gregaria* [8,13,15].

In *P. xylostella*, dsRNases were mainly expressed in the intestinal tract and hemolymph. DsRNases were mainly expressed in the head and intestine in *T. castaneum* [23], and in the intestines and salivary glands in *Halyomorpha halys* [24]. Therefore, dsRNase expression is inconsistent in different insects. The ability of total protein of hemolytic lymph to degrade dsRNA was higher than that of the intestinal juice in *P. xylostella*. Similar cases are reported in Lepidoptera and Coleoptera [25]. Expression of dsRNases, both in the intestinal tract and hemolymph, might be the reason why the injection of dsRNA is more effective than feeding dsRNA for RNAi in insects [2,25,26,27].

Not all dsRNases are always involved in the RNAi process in insects. PxdsRNase1/ PxdsRNase2/ PxdsRNase3 affected RNAi efficiency, and PxdsRNase4 without signal peptide had no effect on RNAi efficiency, neither through injection or oral intake of dsRNA. Similar case happens in *S. litura*, where four dsRNases could degrade dsRNA and the other one without signal peptide is not active [18]. Only LmdsRNase2 decreased the RNAi efficiency in *L. migratoria* [15], only dsRNase2 in *S. gregaria* [13,28], and only dsRNase3 in *Cylas puncticollis* [29]. Therefore, the involvement of dsRNases in insects is species-specific. In addition to dsRNases, an *RNAi efficiency-related nuclease* (REase) is found to degrade dsRNA and suppress RNAi response in the Asian corn borer, *Ostrinia furnacalis* [19]. Uncovering the molecular mechanisms behind the phenomenon that some factors inhibit the initiation of RNAi responses is imperative to apply RNAi for the control of pests [30,31,32]. In our research, dsPxdsRNase4 did not suppress the expression *PxdsRNase4*, but *PxdsRNase1* in oral experiment. This phenomenon is also found in the Asian migratory locust [13]. It is not clear whether similar situation will occur in other species and the mechanism behind this phenomenon is also uncovered.

PxdsRNase1 degraded dsRNA rapidly, PxdsRNase3 cleaved dsRNA without complete degradation, and PxdsRNase2 could not degrade dsRNA. DsRNase activity depends on the pH of the working environment [13,15]. LmdsRNase1 could degrade dsRNA efficiently under the pH of 5, and could be intensively suppressed at the physiological pH of hemolymph (7.0), leading to the long-term stability of dsRNA in the hemolymph [15]. In addition, the length of dsRNA could also affect dsRNase’s activity [7]. Therefore, the effect of factors, including pH and the length of dsRNA on the activity of PxdsRNase, need to be investigated in the future to understand the stability of dsRNA in *P. xylostella*. In the meantime, new techniques, such as nanoparticles [33,34], transfection reagents [35], and dsRNA encapsulation using bacteria [36] are currently under the investigation by many groups for using RNAi technology as a new method for pest control. It is promising for RNAi-based pest control after the mechanism of dsRNase is explored.

## 5. Conclusions

We found four PxdsRNases, and three of them, PxdsRNase1, PxdsRNase2, and PxdsRNase3 were verified to be involved in the RNAi process in *P. xylostella*. In vitro, the recombinant protein of PxdsRNase1 degraded dsRNA completely, and PxdsRNase3 cleaved dsRNA without complete degradation. This study provided a fundamental basis for understanding the mechanism of dsRNase involvement in the RNAi process and using RNAi to control *P. xylostella* in the future.

## Figures and Tables

**Table 1:** Primers used in the experiment.

Use of Primers	Gene	Primer Sequence (5′-3′)
cDNA cloning	*PxdsRNase1*	F: ATGCTTCACAAATGGGTTCTAATG
		R: TCACAGTAGTAATCCAGTGACTTTG
	*PxdsRNase2*	F: ATGATGTTGCGTGGTTGTGTGC
		R: TTAAACTAAAAGCCCATTCACGGTAAAAG
	*PxdsRNase3*	F: ATGCTGCGTCCTTGTCTGC
		R: TTAAGCCAACAGCCCATTAACTTTG
	*PxdsRNase4*	F: ATGATTCACAAAAAACTTTTACAAG
		R: TTACACTTTCTTTCCGTTGATGCTAG
qPCR primer	*PxdsRNase1*	F: GCGCGAATTGTACCCTCTGT
		R: GGACTCAGCAGCCAATCAAC
	*PxdsRNase2*	F: GGGCGTATGGAGTCTTCCTG
		R: CGAACTCCTCTTCTCCCAGC
	*PxdsRNase3*	F: CGGACACTCCTTCCACGAC
		R: TGTAGCCGACCCTGATGACC
	*PxdsRNase4*	F: AAGTAGCCCAACTCAGTGCC
		R: AGCAGACACGGTCCCGAATA
	*EF1*	F: GCCTCCCTACAGCGAATC
		R: CCTTGAACCAGGGCATCT
	*PxCht*	F: AGACTTGATGGTGTTCGCGT
		R: GTCCACCTTCTGCCCTATCG
dsRNA primer	dsPxdsRNase1	F: TAATACGACTCACTATAGGGTAGATGCTCCTCACCTCCA
		R: TAATACGACTCACTATAGGGTCTCTCCGAACGCAAACAC
	dsPxdsRNase2	F: TAATACGACTCACTATAGGGGTGGAGGATGGTAAAGCGAC
		R: TAATACGACTCACTATAGGGGTGGCGAACACAAAGTCCGT
	dsPxdsRNase3	F: TAATACGACTCACTATAGGGTCCAAGACTGTGGCTACTGC
		R: TAATACGACTCACTATAGGGGTCAGATGCCCTCGGTTCAA
	dsPxdsRNase4	F: TAATACGACTCACTATAGGGCGGGTTCCTAACTGGGTGTT
		R: TAATACGACTCACTATAGGGCCACCGAGTTGCCTCCTATC
	dsPxCht	F: TAATACGACTCACTATAGGGAACGAAAAGGTCTGGATCTG
		R: TAATACGACTCACTATAGGGGGGTGGTCTCAGCCTAACAT
	dsGFP	F: TAATACGACTCACTATAGGGGCTTCTCGTTGGGGTCTTTG
		R: TAATACGACTCACTATAGGGACCACATGAAGCAGCACGAC

Note: The underline sequence represents the sequence of T7 promoter.

## Data Availability

Data are available from the article and from authors on request.
